# The Effect of Acyclic Retinoid on the Metabolomic Profiles of Hepatocytes and Hepatocellular Carcinoma Cells

**DOI:** 10.1371/journal.pone.0082860

**Published:** 2013-12-23

**Authors:** Xian-Yang Qin, Feifei Wei, Masaru Tanokura, Naoto Ishibashi, Masahito Shimizu, Hisataka Moriwaki, Soichi Kojima

**Affiliations:** 1 Micro-signaling Regulation Technology Unit, RIKEN Center for Life Science Technologies, Wako, Saitama, Japan; 2 Department of Applied Biological Chemistry, Graduate School of Agricultural and Life Sciences, University of Tokyo, Tokyo, Japan; 3 Tokyo New Drug Research Laboratories, Pharmaceutical Division, KOWA Company, Ltd., Tokyo, Japan; 4 Department of Gastroenterology, Gifu University Graduate School of Medicine, Gifu, Japan; 5 Japan Society for the Promotion of Science, Tokyo, Japan; University of Navarra School of Medicine and Center for Applied Medical Research (CIMA), Spain

## Abstract

**Background/Purpose:**

Acyclic retinoid (ACR) is a promising chemopreventive agent for hepatocellular carcinoma (HCC) that selectively inhibits the growth of HCC cells (JHH7) but not normal hepatic cells (Hc). To better understand the molecular basis of the selective anti-cancer effect of ACR, we performed nuclear magnetic resonance (NMR)-based and capillary electrophoresis time-of-flight mass spectrometry (CE-TOFMS)-based metabolome analyses in JHH7 and Hc cells after treatment with ACR.

**Methodology/Principal Findings:**

NMR-based metabolomics revealed a distinct metabolomic profile of JHH7 cells at 18 h after ACR treatment but not at 4 h after ACR treatment. CE-TOFMS analysis identified 88 principal metabolites in JHH7 and Hc cells after 24 h of treatment with ethanol (EtOH) or ACR. The abundance of 71 of these metabolites was significantly different between EtOH-treated control JHH7 and Hc cells, and 49 of these metabolites were significantly down-regulated in the ACR-treated JHH7 cells compared to the EtOH-treated JHH7 cells. Of particular interest, the increase in adenosine-5′-triphosphate (ATP), the main cellular energy source, that was observed in the EtOH-treated control JHH7 cells was almost completely suppressed in the ACR-treated JHH7 cells; treatment with ACR restored ATP to the basal levels observed in both EtOH-control and ACR-treated Hc cells (0.72-fold compared to the EtOH control-treated JHH7 cells). Moreover, real-time PCR analyses revealed that ACR significantly increased the expression of pyruvate dehydrogenase kinases 4 (PDK4), a key regulator of ATP production, in JHH7 cells but not in Hc cells (3.06-fold and 1.20-fold compared to the EtOH control, respectively).

**Conclusions/Significance:**

The results of the present study suggest that ACR may suppress the enhanced energy metabolism of JHH7 cells but not Hc cells; this occurs at least in part via the cancer-selective enhancement of PDK4 expression. The cancer-selective metabolic pathways identified in this study will be important targets of the anti-cancer activity of ACR.

## Introduction

Hepatocellular carcinoma (HCC) represents approximately 85% of all primary liver cancers and is one of the most common malignancies worldwide, especially in Eastern Asia [1]. The prognosis of HCC remains very poor; this poor prognosis is due in part to its high rate of recurrence after initial treatment, which reaches approximately 70% within 5 years [2]. Acyclic retinoid (ACR), a synthetic retinoid with a vitamin A-like structure, prevents the recurrence and development of HCC in patients after the surgical removal of primary tumors [3], [4]. ACR is currently undergoing phase II/III clinical trials (JapicCTI-121828) in Japan and is expected to become the first chemopreventive agent.

Another important characteristic of ACR is that it selectively suppresses the growth of HCC cells (JHH7 and others) but not normal hepatic cells (Hc) [5], [6]. Although the mechanism underlying this effect is not fully understood, previous basic and clinical studies by our group and others have suggested that both non-genomic and genomic signaling pathways may be responsible for the cancer-selectivity of ACR [5], [7], [8], [9], [10], [11], [12]. A typical example is the prevention by ACR of the aberrant hyper-phosphorylation and inactivation of retinoid X receptor (RXR) α that occurs during carcinogenesis in HCC [12] and the subsequent induction of apoptosis in HCC cells by the restoration of the expression of RXRα downstream genes such as p21 [11], transglutaminase 2 (TG2) [5] and more. However, to the best of our knowledge, no information is available regarding the effect of ACR on the metabolism of HCC cells.

Recently, the approach of targeting cancer metabolism to develop and improve cancer therapeutics has received a great deal of attention [13]. A distinguishing feature of cancer is that the metabolic pathways of cancer cells are adapted to support rapid and uncontrolled cell proliferation. One of the best-known alterations in cancer cell metabolism is a switch from mitochondrial oxidative phosphorylation to cytoplasmic glycolysis; this switch is known as the Warburg effect [14]. It is possible that targeting cellular metabolism may suppress cancer. In fact, several metabolism-targeting therapies have been already proven to be effective in the treatment of diverse human tumors [13], [15].

Although chronic hepatitis B virus (HBV) or hepatitis C virus (HCV) infections are believed to account for approximately 80% of HCC [16], a growing body of evidence indicates that metabolic syndrome is also a risk factor for the development of HCC [17]. Indeed, it is extremely difficult to find a single essential target for cancer therapeutics, due to the remarkable heterogeneity and adaptability of cancer cells. It is likely that further investigations into the effect of ACR on cancer cell metabolism will improve our understanding of the molecular pathways underlying the cancer-selective growth suppressive effect of ACR and benefit the development of more effective cancer drugs and therapies against HCC. To achieve this, both nuclear magnetic resonance (NMR)-based and capillary electrophoresis time-of-flight mass spectrometry (CE-TOFMS)-based metabolome analyses were performed in JHH7 and Hc cells after treatment with ACR.

## Materials and Methods

### Materials

ACR (NIK-333) was supplied by Kowa Co. Ltd. (Tokyo, Japan). All-*trans*-retinoic acid (AtRA) was purchased from Sigma-Aldrich (St Louis, MO, USA). Ethanol (EtOH) was obtained from Wako Industries (Osaka, Japan), and used as the primary solvent for all reagents. EtOH solutions were further diluted into cell culture media for treatments. The final concentration of EtOH in media used as a control was 0.05% (vol/vol).

### Cell culture

The JHH7 HCC cell line was kindly supplied by Dr. Matsuura (Jikei University School of Medicine, Tokyo, Japan) [18]. The normal human hepatocyte cell line (Hc) was purchased from Cell Systems (Kirkland, WA, USA). Both cell lines were maintained in Dulbecco's Modified Eagle Medium (DMEM; Wako Industries) containing 10% fetal bovine serum (FBS, Mediatech, Herndon, VA, USA), 100 U/ml penicillin/streptomycin and 2 mmol/L L-glutamine (Mediatech, Herndon, VA, USA) and grown at 37°C in a humidified 5% CO_2_ incubator. For chemical treatment, the cells were cultured in serum-free media containing EtOH or ACR at the appropriate concentrations.

### NMR-based metabolomics

For NMR analyses, cells (approximately 1×10^7^ cells) treated with EtOH control or 10 μM ACR control for 4 h or 18 h were harvested by scraping as previously described [19]. The one-dimensional (1D) ^1^H spectra were measured at 500 MHz on a Varian Unity INOVA-500 spectrometer. All NMR spectra were processed using the MestReNova program (Version 5.3.0, MestRec, Santiago de Compostela, Spain). Metabolites were identified using publicly accessible databases, including BioMagRes data bank (http://www.bmrb.wisc.edu), the Metabolomics Database of Linkoping (http://www.mdl.imv.liu.se), and the Human Metabolome Data Bank (http://www.hmdb.ca). Detailed NMR methods have been described previously [19], [20].

### CE-TOFMS analyses

JHH7 and Hc cells (approximately 5×10^6^ cells) treated with EtOH control or 10 μM ACR for 24 h were washed twice with a 5% mannitol solution, and then 1,300 μL of a methanol solution containing 10 μM internal standards was added. Metabolome extraction was then performed as previously described [21]. The metabolic profiles of the cells were then measured using a CE-TOFMS-based metabolomics technique, which is a novel strategy for analyzing and differentially displaying metabolic profiles [21]. CE-TOFMS was carried out using an Agilent CE Capillary Electrophoresis System equipped with an Agilent 6210 Time-of-Flight mass spectrometer, Agilent 1100 isocratic HPLC pump, Agilent G1603A CE-MS adapter kit, and Agilent G1607A CE-ESI-MS sprayer kit (Agilent Technologies, Waldbronn, Germany).

### Data analysis for CE-TOFMS and metabolite identification

The raw data obtained by CE-TOFMS were analyzed using KEIO MasterHands software exactly as previously described [22], [23]. Briefly, the injected volume for CE and the sensitivity of MS were corrected using internal standards, and then all the annotated metabolites were further corrected to the same chemicals in a standard mixture to overcome different ionization patterns. The peaks were identified based on the matched mass-to-charge ratio (*m/z*) values and normalized migration times of the corresponding standard compounds.

### Real-time RT-PCR

For PCR analyses, RNA was isolated from each cell culture treated with EtOH, AtRA or ACR for 4 h using an RNeasy Kit (Qiagen, Valencia, CA, USA), and the amount and purity of the isolated RNA were evaluated using a NanoDrop spectrophotometer (NanoDrop products, Wilmington, DE, USA). cDNA was then synthesized using a PrimeScript RT Master Mix Kit (TaKaRa Bio, Otsu, Japan). Oligonucleotide primers were designed using OligoPerfect Designer software (Invitrogen, Carlsbad, CA, USA; http://www.tools.invitrogen.com) and synthesized by Invitrogen. The sequences of the primers and the full gene names are summarized in Table S1. PCR reactions were performed using a the Thermal Cycler Dice™ Real Time System (TP8000; Takara Bio) with SsoAdvanced^TM^ SYBR® Green Supermix (Bio-Rad Laboratories, Hercules, CA, USA).

### Western blot analysis

JHH7 and Hc cells treated with EtOH, AtRA and ACR for 24 h were lysed using RIPA buffer. After boiling at 97°C for 10 min, the protein samples were resolved by sample buffer for sodium dodecyl sulfate (SDS) polyacrylamide gel electrophoresis, run on a 10% gel and transferred to a polyvinylidene difluoride membrane (Bio-Rad Laboratories). The membranes were blocked with 5% nonfat dry milk in Tris-buffered saline (TBS) and 0.1% Tween and then probed with primary antibodies against pyruvate dehydrogenase kinase 4 (PDK4; sc-14492; 1∶1,000 dilution, Santa Cruz Biotechnology, CA, USA), pyruvate dehydrogenase (lipoamide) alpha 1 (PDHA1; sc-377092; 1∶1,000 dilution, Santa Cruz Biotechnology), phospho-PDHA1 (ab92696; 1∶1,000 dilution, Abcam) or Lamin B1 (ab16048; 1∶5,000 dilution, Santa Cruz Biotechnology). The blots were then incubated with horseradish peroxidase-conjugated anti-goat, anti-mouse or anti-rabbit secondary antibodies and detected using the Amersham ECL PlusTM Western Blotting Detection System (GE Healthcare UK, Buckingham, England). Immunoreactive bands were quantified using ImageJ densitometry software (National Institutes of Health, Bethesda, MD), and normalized; the density of the corresponding band in the EtOH control was set to 1.0.

### RNA interference

An siRNA targeting human PDK4 (sc-39030) and a control siRNA (sc-37007) were purchased from Santa Cruz Biotechnology. JHH7 cells were plated in either 96-well plates (1×10^4^ cells/well) for cell proliferation analysis and RNA isolation or 60-mm dishes (3.5×10^5^ cells/dish) for ATP assays 1 day prior to transfection. The cells were then transfected with 50 nM or 100 nM siRNAs using Lipofectamine 2000 (Life Technologies, Grand Island, NY, USA).

### ATP assay

The cellular levels of ATP were measured using a firefly bioluminescence assay kit (AMERIC-ATP kit, Wako Industries) according to the manufacturer's instructions. The luciferase activity was measured using a plate reader (ARVO MX, Perkin Elmer Inc., MA, USA).

### Cell viability assay

The number of viable cells was determined using the Cell Counting Kit-8 (Dojindo Molecular Technologies, Tokyo, Japan) as previously described [5].

### Network generation and pathway analyses

The Ingenuity Pathways Analysis (IPA) program (Ingenuity Systems, Mountain View, CA, USA; http://www.ingenuity.com) was used to identify networks and canonical pathways as previously described [24]. The generated biological networks were ranked by score, which is the likelihood that a set of genes is found in the networks due to random chance as measured by a Fisher's exact test. The resulting canonical pathways were ranked by *P* values, which were calculated using a Fisher's exact test by comparing the number of user-specified genes of interest that participate in a given function or pathway, relative to the total number of occurrences of these genes in all the functional/pathway annotations stored in the Ingenuity Pathways Knowledge Base [25].

### GEO data mining

The normalized PDK4 expression from a clinical data set, which contains transcriptome profiling of 268 HCC tumor, 243 adjacent non-tumor, 40 cirrhotic and 6 healthy liver samples, was downloaded from the Gene Expression Omnibus (www.ncbi.nlm.nih.gov/geo, accession no. GSE25097).

### Statistical and multivariate analyses

All the experiments in this study were performed independently two or more times to ensure the reproducibility of the results. Quantitative data were expressed as the means ± SEMs. The statistical significance of differences between values was assessed using a two-tailed Student's *t*-test or a Mann-Whitney U test. Values of *P*<0.05 were considered to indicate statistical significance. Unsupervised principal component analysis (PCA) was run using SIMCA-P+ software (Version 12.0, Umetrics, Umeå, Sweden).

## Results

### The effect of ACR on the metabolism of JHH7 cells detected using ^1^H-NMR

First, NMR-based metabolomics was performed to investigate the effect of ACR treatment on the metabolism of JHH7 cells. As shown in Figure S1, PCA analysis of the NMR spectra indicated that treatment with ACR for 4 h had a very minor effect on the metabolism of JHH7 cells, while obvious changes were observed after 18 h of ACR treatment compared to the EtOH control.

### Differences between the metabolic profiles of JHH7 and Hc cells treated with EtOH and ACR detected using CE-TOFMS

**Figure 1:**
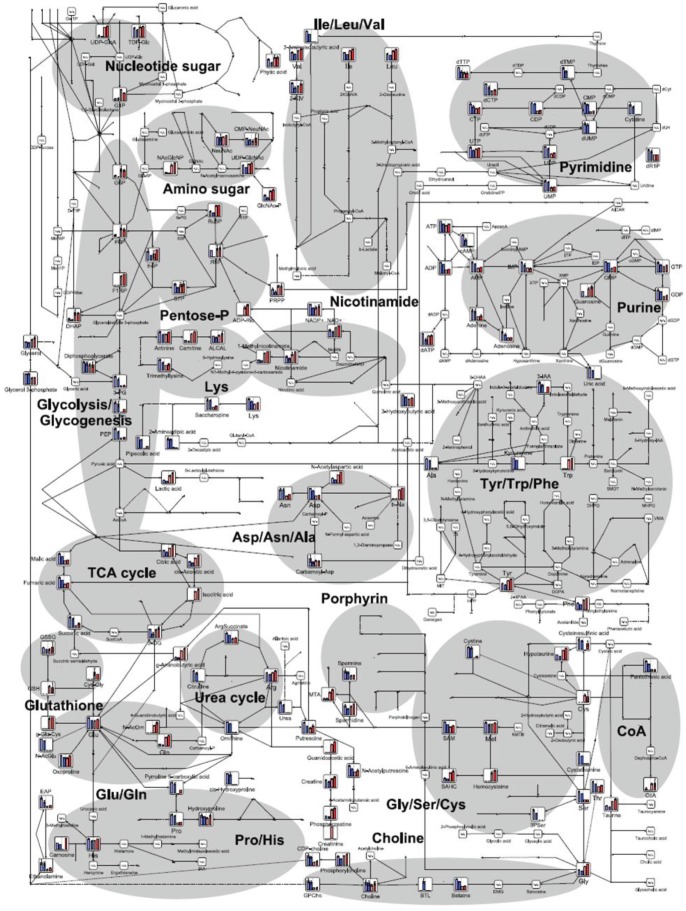
Metabolites in the principle metabolic pathways of EtOH- or ACR-treated JHH7 and Hc cells detected by CE-TOFMS. The relative quantities of the detected metabolites are represented as bar graphs (from left to right: EtOH-treated JHH7, ACR-treated JHH7, EtOH-treated Hc, and ACR-treated Hc). N.D., not detected.

**Figure 2:**
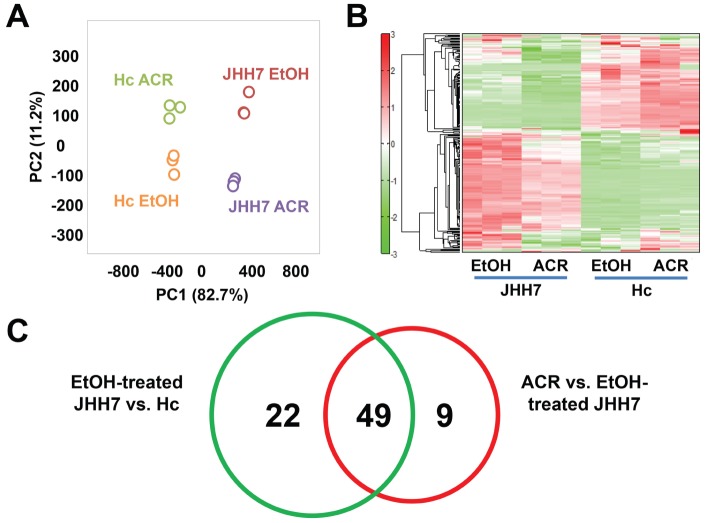
A comparison of the metabolic profile of EtOH- or ACR-treated JHH7 and Hc cells determined by CE-TOFMS. PCA scoreplot (***A***) and heat map (***B***) from metabolic data of JHH7 and Hc cells treated with EtOH and ACR (*n* = 3). Venn-diagrams (***C***) showing the number of metabolites that were significantly deregulated between the two groups.

To further investigate the cancer-selective effect of ACR, the metabolic profiles of JHH7 and Hc cells treated with EtOH and ACR for 24 h was measured using CE-TOFMS analysis. A total of 229 peaks (109 cationic and 120 anionic) were detected in either JHH7 or Hc cells; from these 229 peaks, 88 principal metabolites were quantified (Table S2). The metabolic pathways of all the detected metabolites are illustrated in Figure 1. These metabolites are associated with glycolysis/gluconegenesis, the pentose phosphate pathway, the tricarboxylic acid cycle, the urea cycle, pyrimidine metabolism, nicotinate and nicotinamide metabolism and amino acid metabolism. The result of the comparison of the metabolic profiles of the cells is provided in Figure 2. PCA analysis revealed a very clear distinction between the abundance of intracellular metabolites of JHH7 and Hc cells with and without ACR treatment (Figure 2A), while the first component (PC1) indicated that 67% of the total variance is due to the difference between JHH7 and Hc cells. PC2 (11.2%) indicated that the ACR-treated JHH7 cells have a metabolic profile that is similar to that of the EtOH-treated Hc cells. Furthermore, heatmap analyasis indicated that the metabolic pattern of JHH7 cells was almost completely opposite that of the Hc cells; a similar difference was observed between the ACR-treated and EtOH-treated JHH7 cells (Figure 2B). Finally, the cellular content of 71 metabolites in JHH7 and Hc cells was significantly different with *P* values less than 0.05 and fold changes greater than 1.2; 58 metabolites were significantly down-regulated by ACR in JHH7 cells compared to the EtOH control. Forty-nine common metabolites were shared between the two groups (Figure 2C).

### Network generation and pathway analyses

**Figure 3:**
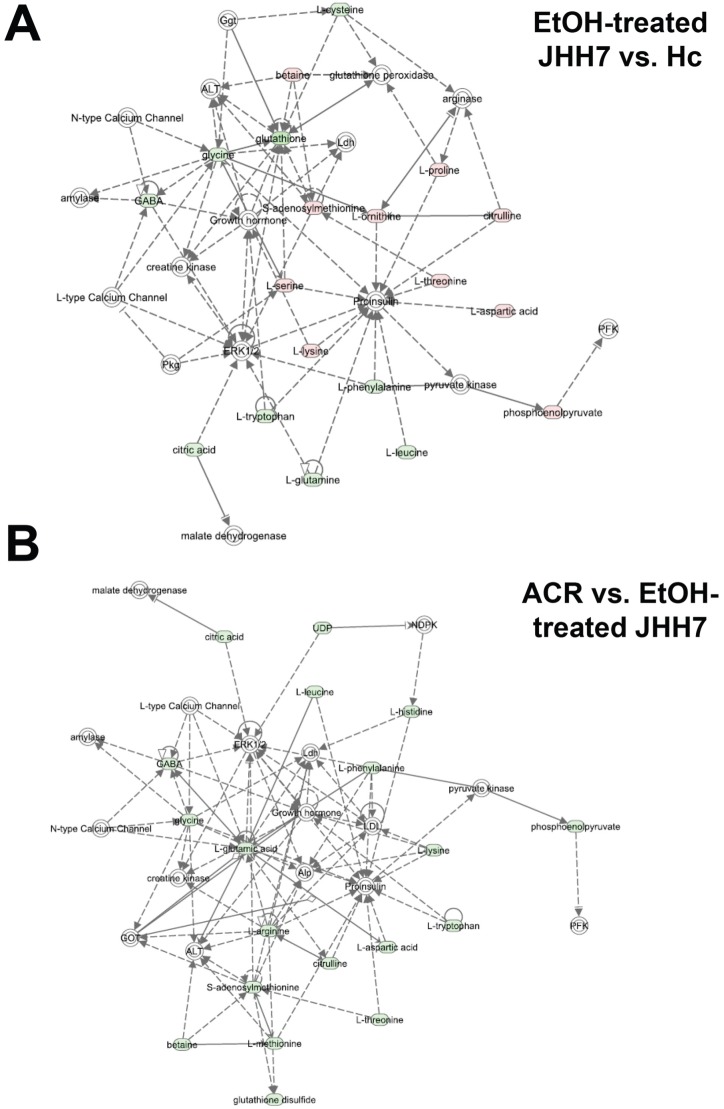
Network generation using Ingenuity Pathway Analysis. The “Increased Levels of Albumin, Amino Acid Metabolism, Molecular Transport” network was associated with metabolites that were significantly different between JHH7 and Hc cells (***A***) and the metabolites that were differentially regulated by ACR in JHH7 cells (***B***). Up-regulated metabolites are indicated in red, down-regulated metabolites indicated in green, and metabolites that were not annotated in this study but are part of this network are indicated in white. Direct relationships are drawn with solid arrows, and indirect relationships are drawn with dashed arrows.

Next, the list of the significantly different metabolites was imported into the IPA platform to investigate possible biological interactions. The biological functions of the top five IPA-generated networks and top five canonical metabolic pathways are summarized in Tables 1 and 2, respectively, and shown in Figure 3. Interestingly, IPA analysis indicated that the most highly populated biological network (“Increased Levels of Albumin, Amino Acid Metabolism, Molecular Transport”) and the top two canonical metabolic pathways (“tRNA Charging” and “Purine Nucleotides De Novo Biosynthesis II”) that were associated with the ACR-regulated metabolites by in JHH7 cells were the same as the networks that were associated with metabolic differences between JHH7 and Hc cells.

**Table 1:** Top five associated network functions generated by IPA.

	Top function	Score
EtOH-treated JHH7 vs. Hc	Increased Levels of Albumin, Amino Acid Metabolism, Molecular Transport	39
	Cellular Growth and Proliferation, Organismal Development, Cellular Compromise	21
	Cardiovascular System Development and Function, Organ Development, Carbohydrate Metabolism	18
	Cellular Growth and Proliferation, Organismal Development, Small Molecule Biochemistry	16
	Carbohydrate Metabolism, Cell Morphology, Cell-To-Cell Signaling and Interaction	11
ACR vs. EtOH-treated JHH7	Increased Levels of Albumin, Amino Acid Metabolism, Molecular Transport	38
	Carbohydrate Metabolism, Molecular Transport, Small Molecule Biochemistry	23
	Cellular Growth and Proliferation, Organismal Development, Small Molecule Biochemistry	16
	Free Radical Scavenging, Small Molecule Biochemistry, Molecular Transport	14
	Post-Translational Modification, Cellular Assembly and Organization, Developmental Disorder	6

**Table 2:** Top canonical pathways identified by IPA.

	Top canonical pathway	P-Value
EtOH-treated JHH7 vs. Hc	tRNA Charging	6.82E-30
	Purine Nucleotides De Novo Biosynthesis II	1.30E-24
	Pyrimidine Ribonucleotides De Novo Biosynthesis	9.94E-20
	Superpathway of Citrulline Metabolism	9.93E-19
	Gluconeogenesis I	8.21E-18
ACR vs. EtOH-treated JHH7	tRNA Charging	9.48E-30
	Purine Nucleotides De Novo Biosynthesis II	2.91E-19
	Arginine Biosynthesis IV	3.10E-15
	Citrulline-Nitric Oxide Cycle	1.84E-14
	NAD biosynthesis II (from tryptophan)	5.72E-14

### ACR inhibits the increase in adenosine-5′-triphosphate (ATP) production in JHH7 cells

**Figure 4:**
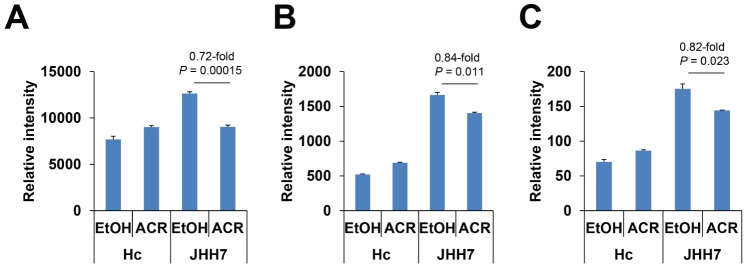
Levels of adenosine nucleotides in EtOH or ACR-treated JHH7 and Hc cells determined by CE-TOFMS. ATP (***A***), ADP (***B***) and AMP (***C***) levels.

A comparison of the biosynthetic metabolites (nucleotides, amino acids and lipids) in the EtOH- or ACR-treated JHH7 and Hc cells determined by CE-TOFMS is summarized in Table 3. Of particular interest, the changes in the concentrations of adenosine nucleotides are shown in Figure 4. Notably, ATP levels were 1.6-fold higher in the EtOH-treated JHH7 cells than in the EtOH-treated Hc cells; ACR suppressed this increase, nearly to the basal levels observed in Hc cells (0.72-fold and *P* = 0.00015 compared to the EtOH-treated JHH7 cells). In contrast, only a very minor effect of ACR was observed on the levels of adenosine diphosphate (ADP) and adenosine monophosphate (AMP) in JHH7 cells (0.84- and 0.82-fold compared to the EtOH control, respectively).

**Table 3:** Comparison of biosynthetic metabolites in EtOH or ACR-treated JHH7 and Hc cells determined by CE-TOFMS.

Biosynthetic pathways	Metabolites	KEGG	Fold change			
			EtOH-treated JHH7 vs. Hc		ACR vs. EtOH-treated JHH7	
			Mean	SEM	Mean	SEM
Nucleotide biosynthesis	AMP	C00020	2.50	0.10	0.82	0.00
	ATP	C00002	1.64	0.02	0.72	0.01
	CTP	C00063	1.84	0.11	0.74	0.05
	dATP	C00131	1.26	0.02	0.47	0.07
	dCTP	C00458	2.74	0.06	0.69	0.07
	dTTP	C00459	1.96	0.04	0.61	0.06
	GDP	C00035	2.76	0.15	0.82	0.02
	GTP	C00044	1.67	0.11	0.74	0.04
	IMP	C00130	2.33	0.11	0.71	0.08
	PRPP	C00119	0.68	0.08	0.53	0.11
	Ribulose 5-P	C00199	0.46	0.05	0.68	0.05
	UDP	C00015	2.81	0.05	0.82	0.05
	UTP	C00075	1.22	0.04	0.68	0.02
Amino acid biosynthesis	Ala	C00041	9.23	0.31	0.79	0.02
	Asp	C00049	3.23	0.08	0.77	0.01
	Glu	C00025	1.38	0.02	0.76	0.01
	Gly	C00037	0.64	0.02	0.82	0.01
	Ile	C00407	0.80	0.03	0.79	0.01
	Leu	C00123	0.73	0.03	0.78	0.02
	Lys	C00047	1.94	0.06	0.77	0.03
	Phe	C00079	0.69	0.02	0.78	0.01
	Ser	C00065	5.31	0.20	0.81	0.02
	Thr	C00188	1.94	0.07	0.77	0.00
	Trp	C00078	0.06	0.00	0.78	0.02
	Tyr	C00082	0.71	0.03	0.79	0.02
	Val	C00183	0.83	0.04	0.78	0.02
Lipid biosynthesis	3-Hydroxybutyric acid	C01089	1.58	0.03	0.82	0.03
	DHAP	C00111	0.28	0.03	0.52	0.03

### ACR enhances PDK4 expression in JHH7 cells, but not in Hc cells

**Figure 5:**
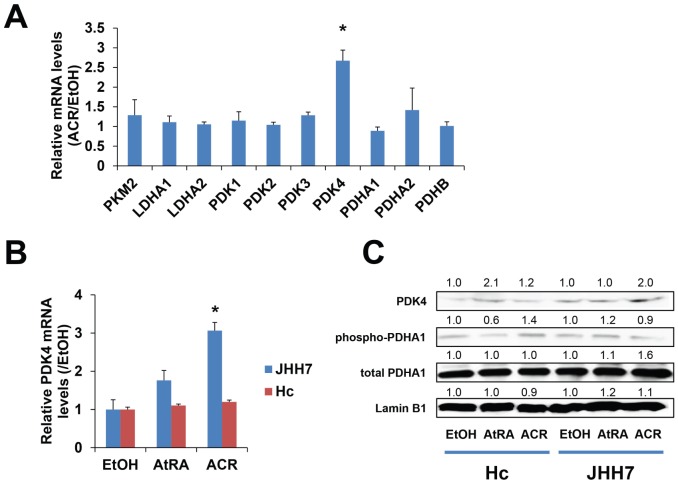
ACR increase the expression of PDK4 in JHH7 cells but not Hc cells. The effect of ACR on the expression of genes related to energy metabolism and ATP production in cancer cells (***A***). Levels of PDK4 mRNA (***B***) and levels of PDH4, phospho-PDHA1 and total PDHA1 protein (***C***) in JHH7 and Hc cells treated with EtOH, AtRA and ACR. The statistical significance of difference was evaluated using the Student's *t*-test.

To further understand the cancer-selective inhibitory effect of ACR on ATP production, a set of genes that is known to be important in the regulation of energy metabolism in cancer cells was selected based on previous reports [26], [27], [28], [29], and the effect of ACR on the expression of these genes was measured using real-time PCR (Figure 5A). Of particular interest, we found that ACR significantly enhanced the expression of PDK4, an important regulator of ATP levels [30], in JHH7 cells but not in Hc cells (3.06-fold; *P* = 0.0033 and 1.20-fold; *P* = 0.062, respectively; Figure 5B). Further western blot analysis revealed a nearly 2-fold increase in PDK4 protein levels after ACR treatment, but ACR did not affect the phosphorylation of PDHA1 in JHH7 cells (Figure 5C).

### Functional analysis of PDK4 in JHH7 cells

**Figure 6:**
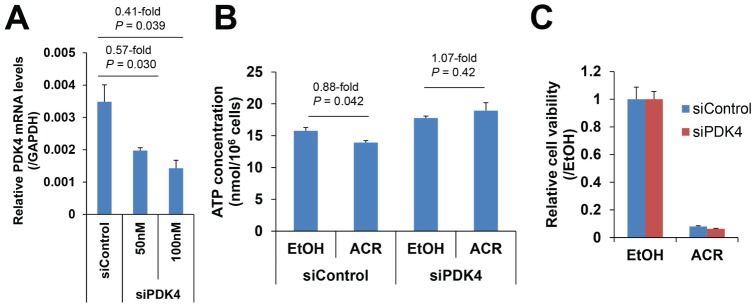
Functional analysis of PDK4 in JHH7 cells. The effect of siPDK4 on PDK4 gene expression (***A***). The effect of 50 nM siPDK4 on the ACR-mediated cellular ATP levels (***B***) and the proliferation (***C***) of JHH7 cells.

Furthermore, loss-of-function experiments were performed to confirm the role of PDK4 in the effect of ACR on cellular ATP levels and the proliferation of JHH7 cells. As shown in Figure 6A, treatment with an siRNA targeting PDK4 (siPDK4) caused a dose-dependent downregulation of PDK4 mRNA expression (0.57-fold and 0.41-fold compared to siControl-treated cells with 50 nM and 100 nM siPDK4, respectively). Interestingly, ACR weakly but significantly inhibited cellular ATP levels in siControl-treated JHH7 cells (0.88-fold and *P* = 0.042 compared with EtOH). In contrast, no significant effect was observed in siPDK4-treated JHH7 cells (1.07-fold and *P* = 0.42 compared with EtOH; Figure 6B). However, PDK4 knockdown did not rescue the inhibitory effect of ACR on the proliferation of JHH7 cells (Figure 6C).

### Clinical expression levels of PDK4

**Figure 7:**
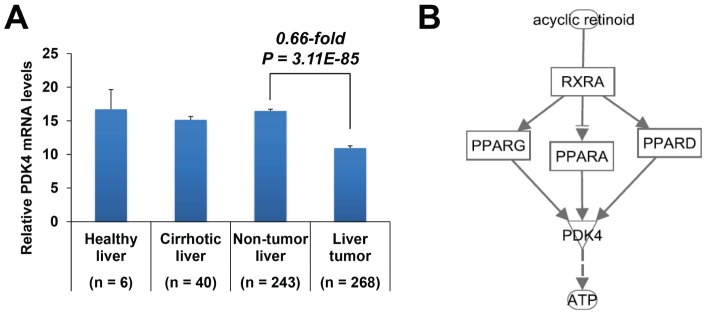
Clinical expression levels of PDK4. The expression of PDK4 mRNA in human liver cancer (GEO data set GSE25097) (***A***). Statistical significance was evaluated using the Mann-Whitney U test. A schematic model of the PDK4-denpendent regulatory network of ACR on ATP production in JHH7 cells generated using IPA (***B***).

The mining of microarray data from a human HCC data set revealed that PDK4 mRNA is significantly down-regulated in liver tumors compared to adjacent non-tumor liver tissues (0.66-fold, *P* = 3.11E-85; Figure 7A). Finally, a PDK4-dependent regulatory network that involves RXR and peroxisome proliferator-activated receptors (PPARs) and summarizes the effects of ACR on ATP production was generated using IPA (Figure 7B).

## Discussion

The war on cancer has continued for more than 40 years, but the gains have been limited. One potential reason for this limited success that typical drug development in oncology has focused on targets that are essential for the survival of all dividing cells, leading to narrow therapeutic windows [31]. Recent advances in metabolite profiling methodologies have generated an alternative window for cancer therapy in targeting cancer metabolism [13]. ACR, a very promising drug that is currently in clinical trials for HCC treatment, has been shown to markedly prevent the recurrence of HCC [3] and selectively inhibit HCC cell growth [5]. We have previously exploited the potential target molecules of ACR that are associated with the promotion of tumor cell proliferation [5] or angiogenesis [32]. In this study, we performed metabolome analyses in JHH7 and Hc cells treated with ACR using both NMR and CE-TOFMS technologies to further understand the molecular pathways that underlie the cancer-selective growth suppressive effect of ACR. We found that ACR selectively suppressed the enhanced nucleotide synthesis and energy metabolism of HCC cells, suggesting that metabolic pathways may be important targets for ACR's anti-cancer activity. The further study of these pathways will benefit the development of more effective cancer drugs and therapies against HCC.

Generally, the metabolic patterns of JHH7 and Hc cells were almost completely opposite to each other (Figure 2), which is consistent with previous reports that cancer cells exhibit considerably different metabolic requirements than most normal differentiated cells and supports the hypothesis that cancer may be a type of metabolic disease [33]. Although the primary cause of cancer is assumed to be at the level of gene expression, metabolites can be considered to be the end products of the cellular regulatory processes that underlie malignant cell growth such as genome instability and mutability [34]. Metabolomic comparison of HCC and control liver tissues have been carried out in several animal and human studies aiming to define metabolomic biomarkers for the early detection of HCC [35], [36]. In this study, the abundance of 71 metabolites was found to be significantly different between JHH7 and Hc cells; 49 of these metabolites were significantly down-regulated by ACR in JHH7 cells (Figure 2 and Table S2). It is not unexpected that an IPA analysis revealed that most of these metabolites are involved in the amino acids and nucleotide biosynthetic pathways, such as “tRNA Charging”, “Purine Nucleotides De Novo Biosynthesis II” and “Pyrimidine Ribonucleotides De Novo Biosynthesis” (Tables 2 and 3). It is well known that cancer cell metabolism must provide a large increase in lipid, protein, and nucleotide synthesis (biomass) to support their uncontrolled high rate of cell growth and proliferation [13]. Our findings indicate that ACR may exert its anti-cancer effect by blocking the biosynthetic processes of cancer cells. Interestingly, a bioinformatics-based anticancer drug screening program in Japan revealed that ACR shares similar anticancer activity pattern with the antipyrimidine drugs doxifluridine and cytarabine and an antipurine drug, 6-mercaptopurine, as assayed by growth inhibition against a panel of 39 human cancer cell lines (JFC39) [37], [38], [39].

ATP is the main energy source for the majority of cellular functions, and impaired cellular energy metabolism is the defining characteristic of nearly all cancers regardless of cellular or tissue origin [33]. Of particular interest, ACR can selectively inhibit the production of ATP in JHH7 cells but not in Hc cells (Figure 4). It has been proven that chemical depletion of ATP can inhibit the growth of HCC cells [40]. This may partially explain the cancer-selective growth suppression effect of ACR. To further understand the molecular signaling mechanisms that underlie this effect, we examined the effect of ACR on the expression of energy production-related genes and observed that the expression of PDK4 was significantly enhanced by ACR in JHH7 cells but not in Hc cells (Figure 5). PDK4 is a key regulator of tricarboxylic acid (TCA) cycle; PDK4 phosphorylates and inactivates the pyruvate dehydrogenase (PDH) complex and thereby switches the energy source for the production of ATP from glucose to fatty acids. Although the cellular pyruvate level was not detected by CE-TOFMS and no effect of ACR was found on the phosphorylation of PDHA1 by western blot analysis (Figure 5C), the knockdown of PDK4 expression using RNA interference in JHH7 cells can rescue the decreased cellular ATP levels induced by ACR (Figure 6B), suggesting that PDK4 may be an important feature of ACR's anti-cancer activity. Moreover, we performed data mining using the GEO database and found that PDK4 expression is significantly down-regulated in liver tumors compared to adjacent non-tumor liver tissues (Figure 7A). The role of PDK4 in cancer therapy is complex; the inhibition of PDK4 is sufficient to inhibit the proliferation of and induce apoptosis in lung cancer cells [41], but the overexpression of PDK4 is also able to decrease ATP levels and suppress de novo lipogenesis and proliferation in breast cancer cells [30]. The specific role of PDK4 in HCC remains to be fully determined. Our results suggest that PDK4 up-regulation has a suppressive effect on HCC. Consistent with this implication, the results of IPA analysis suggest that the cancer-selective, growth-suppressive effect of ACR in inhibiting the ATP production of HCC cells may be related to a putative PDK4-dependent molecular signaling mechanism involving RXR and PPARs, as has been reported in certain fatty acid signaling pathways [42] (Figure 7B).

Although the Warburg effect is a well-recognized hallmark of cancer metabolism, it remains controversial [43]. Warburg hypothesized that tumor cells convert most of their glucose to lactate due to mitochondrial defects. However, subsequent studies showed that most tumor mitochondria are not defective in their ability to carry out oxidative phosphorylation [43]. In fact, we propose that mitochondrial oxidative phosphorylation may be important in supporting HCC cell proliferation based on the following observations: 1) the content of lactate, the major end product of glycolysis, is lower in JHH7 cells than in Hc cells (0.40-fold, Table S2) and 2) ACR up-regulates the expression of PDK4, which attenuates the flux of glycolytic carbon into mitochondrial oxidation and can reduce the production of ATP and inhibit the growth of JHH7 cells.

In summary, our study is the first to investigate the effect of ACR on cancer cell metabolism. A comparison of the metabolic effects of ACR in JHH7 and Hc cells was performed, and a JHH7-selective inhibitory effect of ACR on the production of ATP was observed. The underlying molecular signaling mechanism may relate in part to the cancer-selective enhancement of PDK4 expression, suggesting that mitochondrial oxidative phosphorylation is important in the energy metabolism of HCC cells. However, it should be noted that although PDK4 knockdown can rescue the decreased cellular ATP levels induced by ACR, no effect was observed on the inhibitory effect of ACR on the proliferation of JHH7 cells. Further research is needed to combine the cancer-selective metabolic pathways identified in this study and other signaling pathways to increase our knowledge of ACR's selective anti-cancer activity and to develop more effective cancer drugs and therapies to help us win the war against HCC.

## Supporting Information

Figure S1 **Statistical analysis of metabolites in JHH7 cells detected by ^1^H-NMR.** PCA score plots of the NMR spectra of JHH7 cells treated with EtOH or 10 μM ACR for 4 h and 18 h (***A***) or only 18 h (***B***).
(PPTX)

Table S1 **The primers used in this study.**
(XLSX)

Table S2 **Quantification of major metabolites in JHH7 and Hc cells.**
(XLSX)
